# Evolutionary origin of germline pathogenic variants in human DNA mismatch repair genes

**DOI:** 10.1186/s40246-024-00573-0

**Published:** 2024-01-29

**Authors:** Huijun Lei, Jiaheng Li, Bojin Zhao, Si Hoi Kou, Fengxia Xiao, Tianhui Chen, San Ming Wang

**Affiliations:** 1 Ministry of Education Frontiers Science Center for Precision Oncology, Cancer Centre and Institute of Translational Medicine, Faculty of Health Sciences, University of Macau, Taipa, Macau SAR, 999078 China; 2 Hangzhou Institute of Medicine (HIM), Chinese Academy of Sciences, Hangzhou, 310018 Zhejiang China; 3 Department of Cancer Prevention, Zhejiang Cancer Hospital, Hangzhou, 310022 Zhejiang China

**Keywords:** DNA mismatch repair, Pathogenic variant, Evolutionary origin, Conservation, Ancient genome

## Abstract

**Background:**

Mismatch repair (MMR) system is evolutionarily conserved for genome stability maintenance. Germline pathogenic variants (PVs) in MMR genes that lead to MMR functional deficiency are associated with high cancer risk. Knowing the evolutionary origin of germline PVs in human MMR genes will facilitate understanding the biological base of MMR deficiency in cancer. However, systematic knowledge is lacking to address the issue. In this study, we performed a comprehensive analysis to know the evolutionary origin of human MMR PVs.

**Methods:**

We retrieved MMR gene variants from the ClinVar database. The genomes of 100 vertebrates were collected from the UCSC genome browser and ancient human sequencing data were obtained through comprehensive data mining. Cross-species conservation analysis was performed based on the phylogenetic relationship among 100 vertebrates. Rescaled ancient sequencing data were used to perform variant calling for archeological analysis.

**Results:**

Using the phylogenetic approach, we traced the 3369 MMR PVs identified in modern humans in 99 non-human vertebrate genomes but found no evidence for cross-species conservation as the source for human MMR PVs. Using the archeological approach, we searched the human MMR PVs in over 5000 ancient human genomes dated from 45,045 to 100 years before present and identified a group of MMR PVs shared between modern and ancient humans mostly within 10,000 years with similar quantitative patterns.

**Conclusion:**

Our study reveals that MMR PVs in modern humans were arisen within the recent human evolutionary history.

**Supplementary Information:**

The online version contains supplementary material available at 10.1186/s40246-024-00573-0.

## Introduction

The mismatch repair (MMR) system is essential for DNA damage repair to maintain genome stability. MMR system including *MLH1, MSH2, MSH6* and *PMS2* is conserved from bacteria to eukaryotes [1–4]. Besides the function of individual MMR gene, they can also interact jointly to perform the mismatch repair function. For example, MSH2 and MSH3 can form MSH2-MSH3 dimer and MSH2 and MSH6 can form MSH2-MSH6 dimer to locate the mismatched errors formed during DNA replication for repairing [5, 6], and MLH1 and PMS2 can form MLH1-PMS2 dimer to remove the mismatched bases [7]. In the mismatch repairing process, MSH2-MSH6 (MutS alpha in bacteria) or MSH2-MSH3 complex (MutS beta in bacteria) binds to the dsDNA with mismatched bases, MLH1-PMS2 (MutL alpha in bacteria) then binds to the complex to form a ternary complex to remove the mismatched bases by the activated endonuclease activity of PMS2. The gap is then filled and ligated by DNA polymerase III and DNA ligase [8]. However, MMR genes are vulnerably attached by genetic variation. Functional deficiency of MMR system by the genetic variation leads to uncorrected mismatches, hypermutability and microsatellite instability [9] and a high risk of cancers, mostly colorectal cancer [10] with Lynch syndrome as a typical example [11]. The prevalence of MMR pathogenic and likely pathogenic variant (PV) carriers is estimated being around 3% in colorectal cancer patients and over 0.4% in the general population [12, 13]. The cumulative cancer risk for the carriers of *MLH1, MSH2, MSH6,* and *PMS2* PVs at age 75 is 75.8%, 80.4%, 60.9% and 52.1%, respectively [10].

Pathogenic variation in MMR is often germline [14], highlighting that evolution selection might be involved [12]. Knowledge of the evolutionary origin of human MMR PVs will help to understand the biological basis between MMR variations and hereditary cancer [15]. While the relationship of MMR PVs between humans and other species has been studied, the type of MMR variants analyzed were mostly benign rather than pathogenic [16, 17]. Considering that genetic variation in functionally important genes is often conserved across species, such as the ABO group and major histocompatibility complex (MHC) [18, 19], we hypothesized that human MMR germline PVs might also be related to cross-species conservation as evolutionarily conserved MMR genes. However, the fact that human genetic variations were highly human-specific would also suggest that human MMR germline PVs might arise during the human evolution process [20].

Over the past decades, extensive genomic studies have generated large quantities of genomic sequence data from different species and archaic humans [21–24]. Taking the advantage of the rich resources, we used phylogenetic and anthological approaches to study the evolutionary origin of human PVs in MMR genes. While our phylogenetic study in 99 non-human species across eight clades found no evidence to support cross-species conservation as the source for human MMR PVs, our anthological study tracing human MMR PVs in over 5000 ancient human genomes found extensive sharing of MMR PVs between modern and ancient humans dated within the last 10,000 years.

## Materials and methods

### Data sources

MMR germline variants with annotation information were from the ClinVar database (https://www.ncbi.nlm.nih.gov/clinvar/, accessed on February 13, 2022) [16]. The PVs used for the analyses were those identified as “Pathogenic,” “Likely pathogenic” and “Pathogenic/Likely pathogenic” in the ClinVar database; the benign variants (BVs) were those identified as “Benign,” “Likely benign” and “Benign/Likely benign”; the variants of uncertain significance (VUS) were those identified as “Uncertain significance.” The variants with conflicting interpretations were excluded from the analyses. The genome data for the 100 vertebrates in eight clades of Primate, Euarchontoglires, Laurasiatheria, Afrotheria, Mammal, Aves, Sarcopterygii and Fish were from the UCSC genome browser (http://www.genome.ucsc.edu/, accessed on February 24, 2022). Ancient human genomic information and data were from Allen Ancient DNA Resource (version 50.0, https://reich.hms.harvard.edu/allen-ancient-dna-resource-aadr-downloadable-genotypes-present-day-and-ancient-dna-data, accessed March 18, 2022), European Nucleotide Archive (ENA, https://www.ebi.ac.uk/ena/browser/home, accessed on March 18, 2022) and National Genomic Data Center (NGDC, https://ngdc.cncb.ac.cn/, accessed on April 6, 2022). The original publications of ancient human genomic data and the project accession numbers were listed in the Additional file 2: Table S1.

### Phylogenetic analysis

The reference sequences used for the analyses were human genome hg38, *MLH1* cDNA NM_000249.4, protein NP_000240.1; *MSH2* cDNA NM_000251.3, protein NP_000242.1; *MSH6* cDNA NM_000179.3, protein NP_000170.1; and *PMS2* cDNA NM_000535.7, protein NP_000526.2 [25]. Cross-species sequence alignment was carried out through the UCSC browser comparative genomics alignment pipeline. The phylogenetic tree for the 100 vertebrates was from the UCSC resource (http://hgdownload.cse.ucsc.edu/goldenPath/hg38/multiz100way), where the phastCons and phyloP programs in the Phylogenetic Analysis with Space/Time models (PHAST) package [26] were used to calculate evolutionary conservation scores for each mapped site in the 100 vertebrate species. Both programs employed the same parameters, the unaligned bases and gaps were treated as missing data. Evolutionary tree was constructed by the phyloFit program from the PHAST package [27], with branch length denoting evolutionary distance between species. Additionally, we used a Python-based method (https://github.com/Skylette14/GetBase) to acquire the base information in non-human vertebrates that matched with the human PVs in repeat-masked and aligned genomic sequences. ProteinPaint (https://proteinpaint.stjude.org/) [28] was used for the visualization of shared PVs distributed in the functional domains of the corresponding protein.

### Anthropological analysis

The reference genome sequences from each individual was verified either by referring to the header information of each original file downloaded or the reference genome sequences indicated in the original publications. Only those whose reference genome sequences were hg19, GRCH37 or hs37d5 were included for further analysis. The positions of the four MMR genes were based on hg19 by Ensembl [29]: *MLH1*: chr3:37034823–37107380, *MSH2*: chr2:47630108–47789450, *MSH6*: chr2:47922669–48037240 and *PMS2*: chr7:6012870–6048756. mapDamage (version 2.1.1) was used to assess postmortem damage and to rescale the quality scores of likely damaged positions in ancient genomes [30]. The Mpileup command of the SAMtools was used to call the variants and generate the vcf files with a minimum base quality of 1 [31]. Variants were then annotated using wANNOVA (https://wannovar.wglab.org/) [32]. We used the Position Converter in Mutalyzer3 (https://mutalyzer.nl/) to convert all the variants in each gene to the same sequence identifier, and further checked the results with the Name Checker in Mutalyzer3 to verify that HGVSg corresponds to the correct position [33]. Geographical and chronological information on the ancient human was obtained from related publications. The distribution map of ancient humans sharing modern human MMR PVs was visualized with MATLAB (version R2022a). To ensure the homogenization of the collected ancient genome data, we used only the ancient genome data aligned to the reference human genome sequences to ensure that the data from different sources were consistent regardless their DNA extraction and sequencing conditions. We further used MapDamage program to remove the deamination sequences. A typical feature of the ancient DNA damage is purine-loss fragmentation, which results in the overhanging ends where cytosine deamination is more common than within the double-stranded parts. As such, increased C > T mis-incorporation at read starts and G > A at read ends were high in ancient DNA sequencing data [34]. To avoid the bias, we used the Tablet program (version 1.21.02.08) to visualize the sequence assemblies. The C > T variants located within 2-bp at the read starts and G > A variants within 2-bp at the read ends were excluded.

### Statistical analysis

Kruskal–Wallis test was used to compare the MMR PV numbers shared between different groups, Chi-squared test was used to assess the difference in distribution pattern. Two-side *P* < 0.01 was considered as statistically significant. Statistical analyses were performed in Prism, GraphPad (version 9.0.0).

## Results

### Cross-species conservation of human MMR variants

**Fig. 1:**
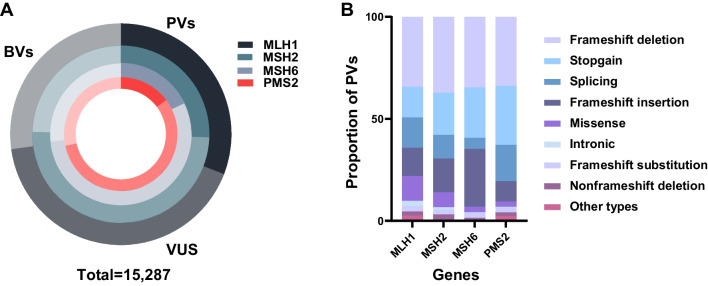
Summary of human MMR variants used in the study. **A** The proportion of MMR PVs, BVs and VUS. **B** Variation types of PVs in each MMR gene

**Fig. 2:**
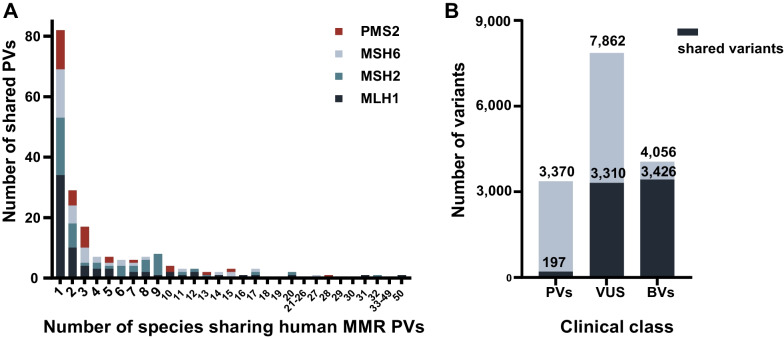
MMR variants shared between human and other species. **A** Summary of all MMR PVs shared between human and non-human species. The color bars show the proportion of each gene in all shared PVs. **B** Differences of MMR PVs, BVs and VUS shared with other species. In contrast to the highly shared BVs and VUS, only a small portion of PVs shared with other species

**Fig. 3:**
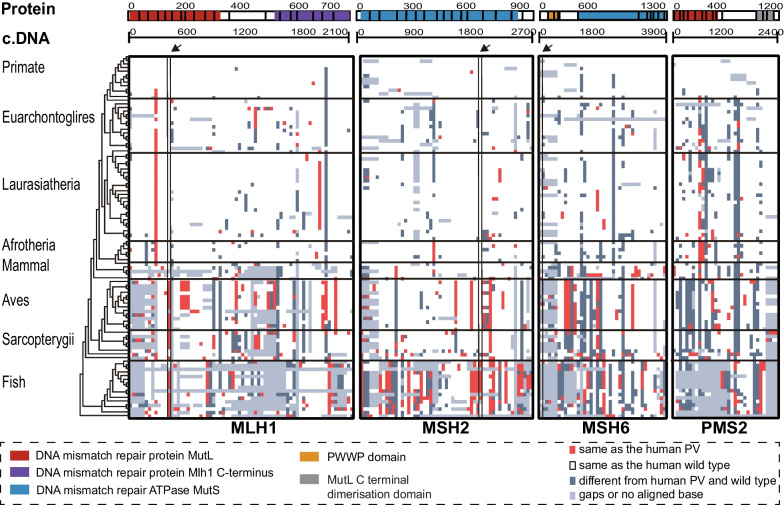
Distribution of human MMR PVs in 100 vertebrates. Each row represents a species and the order is sorted based on phylogenetic tree. Each column represents a locus in an MMR gene. The heatmap displays the 197 MMR PVs shared with non-human vertebrate species. The arrows indicate the founder MMR PVs in modern humans (*MLH1* c.392C > G, *MSH2* c.1906G > C and *MSH6* c.10C > T). The distribution of shared human PVs was compared among clades (Adjusted *P* < 0.0001, two-tailed Kruskal–Wallis test with Benjamini–Hochberg correction)

Overall, 15,287 variants for the four MMR genes of *MLH1, MSH2, MSH6* and *PSM2* were retrieved from the ClinVar database, of which 3369 (22.0%) were classified as PVs, 7892 (51.6%) as VUS and 4056 (26.5%) as BVs (Fig. 1A, Additional file 3: Table S2). To investigate whether the human variants originated from cross-species conservation, we aligned the genomes of 99 non-human vertebrates from eight clades to the human genome to locate the positions conserved between humans and these species (Figs. 2 and 3). The results showed that 197 (5.8%) of the 3369 human MMR PVs including 70 *MLH1* PVs, 55 *MSH2* PVs, 40 *MSH6* PVs and 33 *PMS2* PVs were shared with non-human vertebrates (Fig. 2B). The most shared PV in all four MMR genes was *MLH1* c.208-3C > T with 50 species. Despite the fact that frameshift deletion was the most common type of human PVs (Fig. 1B), stopgain was the most commonly shared PVs (Additional file 4: Table S3A). The shared PVs were distributed in exons and exon-intronic boundaries but not in specific functional domains of MMR genes (*P* > 0.05, Additional file 1: Fig. S1).

A consistent pattern was that the species sharing human MMR PVs were mostly in the clades of Mammal, Aves, Sarcopterygii and Fish distant from humans in the phylogenetic tree. The comparison of shared human PV numbers among eight clades showed a significant difference (The adjusted *P* < 0.0001, Kruskal–Wallis test with Benjamini–Hochberg correction). The closer of the phylogenetic relationship to the humans, the fewer species shared human PVs (Fig. 3, Additional file 4: Table S3B–E). For example, Wallaby (Mammal) had the highest sharing number of 10 human *MSH6* PVs, White-throated sparrow (Ave) had the highest sharing number of 13 human *MLH1* PVs, Xenopus tropicalis (Sarcopterygii) had the highest sharing number of five human *PMS2* PVs, and Princess of Burundi (Fish) had the highest sharing number of 18 human *MSH2* PVs. Few human MMR PVs were shared in Primate, and none were shared in Chimp, Gorilla and Orangutan, the species with the closest phylogenetic relationship with human (Fig. 3, Additional file 4: Table S3B–E). The closest species to human in Primates sharing human PVs was Baboon diverged from human around 30.5 million years ago [35]. It shared human *MLH1* missense PV c.1943C > T, which was shared in Baboon only but not in the other 98 vertebrates (Additional file 4: Table S3). Mouse, rat and zebrafish, which are common models used in cancer research, shared only 4, 3 and 17 human MMR PVs, respectively. We also compared the haplotype-verified human MMR founder PVs in 99 vertebrates [36]. Of the 34 human MMR founder PVs, only three of *MLH1* c.392C > G, *MSH2* c.1906G > C and *MSH6* c.10C > T were shared with non-human species distal from human with the closest shared species of Platypus (Mammal), whereas none were shared in the species in Primate, Euarchontoglires, Laurasiatheria or Afrotheria (Additional file 5: Table S4).

**Fig. 4:**
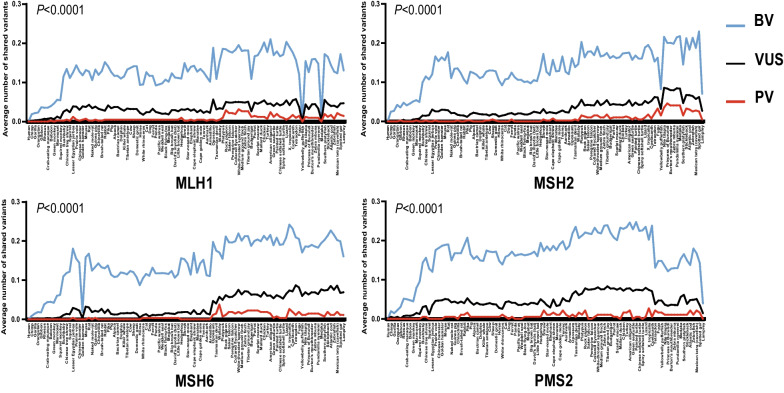
Comparison for the distribution of human MMR PVs, BVs and VUS variants in non-human vertebrates. *X*-axis: sorted species based on the phylogenetic tree (human on the left side of the *X*-axis); *Y*-axis: the number of MMR variants shared between human and non-human vertebrates. The number of shared PVs, VUS and BVs was compared in each gene (Adjusted *P* < 0.0001 in *MLH1, MSH2, MSH6* and *PMS2*, two-tailed Kruskal–Wallis test with Benjamini–Hochberg correction)

We also analyzed the presence of human BVs and VUS in non-human vertebrate species. We observed that 84.5% (3,426/4,056) BVs and 41.9% (3,310/7,892) VUS were present in non-human vertebrates (Fig. 2B). However, the numbers of shared PVs, VUS and BVs in each MMR gene were significantly different (Fig. 4, *P* < 0.0001 in each gene by two-tailed Kruskal–Wallis test with Benjamini–Hochberg correction). The species sharing human MMR BVs and VUS were much closer to humans than PVs (Fig. 4). For example, six human *MLH1* BVs, ten human *MSH2* BVs, one human *MSH6* BV and eight human *PMS2* BVs were shared with chimp in Primate.

The data from phylogenetic analyses demonstrate that human MMR BVs were largely but human MMR PVs were highly unlikely originated from cross-species conservation.

### Archeological analysis of human MMR PVs

**Fig. 5:**
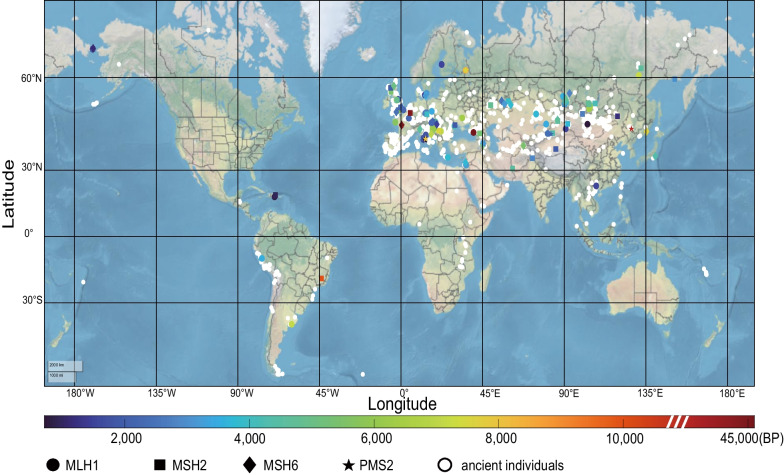
Geographical distribution of the ancient human fossils sharing MMR PVs in modern humans. White dot: ancient fossils identified; colored circles: ancient human individual with *MLH1* PVs; colored squares: ancient human individual with *MSH2* PVs; colored diamonds: ancient human individual with *MSH6* PVs; colored pentagrams: ancient human individual with *PMS2* PVs. The color line at the bottom shows the timing distribution of the ancient fossils sharing MMR PVs in modern humans

We next performed an anthological analysis to test whether human PVs would arise in human history. From publications and databases, we collected comprehensive ancient human genomic data composed of 5064 ancient individuals in six continents dated from 45,045 to 100 years before present (BP). The data also included 29 Neanderthals, three Denisovans, an offspring of a Neanderthal mother and a Denisovan father dated from 130,000 to 37,000 years BP (Additional file 6: Table S5B–E, Fig. 5).

Overall, 121 (3.6%) of 3369 human MMR PVs were identified in 155 ancient humans (Additional file 6: Table S5A-E), including 44 *MLH1* PVs in 63 ancient individuals, 46 *MSH2* PVs in 58 ancient individuals, 18 *MSH6* PVs in 29 ancient individuals and 13 *PMS2* PVs in 16 ancient individuals. The most shared MMR PVs were c.676C > T carried by seven ancient individuals in *MLH1*, c.1165C > T carried by five ancient individuals in *MSH2*, and c.718C > T carried by four ancient individuals in *MSH6*; the oldest shared MMR PV was the stopgain *PMS2* c.400C > T identified in an individual in northeast Asia (Harbin, China) dated to 34,324–32,360 BP [37] and in an individual in Yili, China dated to 2318–2123 BP [38]; the most recently shared PV was *MLH1* c.677G > A in an individual in central Asia (Shunkhlai Mountain, Mongolia) dated to 784–639 BP [39], an individual dated to 2002 BP, three individuals dated to 3950–3650 BP and 4440 BP in Europe (UK, Greece and Hungary), and an individual dated to 7160 BP in South America (Argentina). Overall, the ancient humans sharing the MMR PVs were mainly distributed in Europe, Asia, South America and North America but not in Africa and Oceania (Fig. 5), and 98.3% (119/121) of the PV-sharing carriers were dated within the past 10,000 years. The shared PVs were not clustered in specific function domains of MMR genes (Additional file 6: Table S5B–E).

Nearly all the reported human MMR founder PVs were arisen within the last 2000 years (Additional file 5: Table S4). For example, the Spanish founder *MLH1* c.306 + 5G > A was the oldest one dated to 1879 years ago [40]. We observed that several MMR founder PVs in modern humans were also present in ancient humans. For example, *MSH2* c.1165C > T is a French-Canadian founder PV (its arising time was not determined yet) for Lynch syndrome [41]. The variant was present in five ancient individuals of Europe and West Asia; *MLH1* c.589-2A > G is an American founder PV arose 340–585 years ago [42]. It was identified in two ancient individuals, one dated to 4440–4250 BP in southwest Asia ('Ain Ghazal, Jordan) [43] and the other 1400–900 BP in northeast Asia (Chukotka Autonomous Okrug, Russian) [44].

There was 1 PV in each human MMR gene shared in Neanderthals, all were stopgain (Additional file 6: Table S5B-E). *MLH1* c.1225C > T was identified in a Neanderthal (Mezmaiskaya 2) dated to 44,600–42,960 BP [45], *MSH2* c.1120C > T in a Neanderthal (Goyet Q56-1) dated to 43,000–42,080 BP, *MSH6* c.3772C > T and *PMS2* c.1882C > T in a Neanderthal (Les Cottés Z4-1514) dated to 43,740–42,720 BP. No human MMR PV was identified in Denisovan.

Evidence from our archaeological analyses indicates that MMR PVs in modern humans were originated in recent human history and the extinct Neanderthals also made a partial contribution.

## Discussion

Using both phylogenetic and archeological approaches, our study systematically analyzed the evolutionary origin of germline variation in human MMR genes. Data from our study indicate that human MMR PVs were not originated from cross-species conservation but mostly arose during human evolution in the past 10,000 years, and the extinct Neanderthals also contributed certain MMR PVs in modern humans.

Many human MMR PVs were shared with the species in the clades of other Mammals, Aves, Sarcopterygii and Fish, distal from the humans in the evolution tree. Mouse, rat and zebrafish also shared a few human MMR PVs. While the exact mechanism remains to be determined, the compensation theory was proposed to explain the far-distance sharing of genetic variants, which stated that human PVs could be wild-type in non-human species due to intramolecular compensatory changes in these species [46–49], thus the human MMR PVs present in distal species may not be deleterious as they are in humans. Epistasis offers another explanation that the fitness effect of genetic variants is greatly influenced by the genetic background and beneficial variants are more likely to be epistatic [50].

Data from our study demonstrate that human MMR PVs mostly arose within the last 10,000 years. The timing was concurrent with the rapid population growth of the modern human population following a demographic bottleneck after the withdrawal of the last glacial period [51]. Although strong deleterious variations harmful to survival and reproduction are expected to be eliminated rapidly, mildly deleterious variations may be more tolerable [52, 53]. This is consistent with our previous observation that the Ka/Ks ratio was 0.83 in *MLH1*, 0.92 in *MSH2*, 1.07 in *MSH6* and 1.17 in *PMS2* [54]. Alternatively, the time was not long enough to allow evolution selection to function. The lethality of germline MMR PVs mainly causes high cancer risk in the carriers post reproduction age [10]. As such the PVs were already transmitted during the reproduction age to the next generation. In addition, environmental factors like refined diet, lack of exercise and obesity in modern society were not prevalent among ancient humans living in gathering or agricultural conditions but become epidemic nowadays [55]. MMR deficiency can greatly increase genetic variation and diversity [56, 57], and enhance the survival of unicellular organisms [57–59]. While MMR variation can lead to dysregulation of cell growth in structured complex organisms, it may provide an adaptive benefit for the populations facing environmental stress [59–62].

Neanderthals and Denisovans are extinct hominins. Their genomic admixture with modern humans has been linked to multiple physiological features and disease susceptibility in modern humans, including pigmentation, immunity, metabolism, cognition traits, coronary artery disease and albumin/globulin ratio, and COVID-19 susceptibility [63–66]. The presence of human MMR PVs in Neanderthals highlights that hominins may also contribute to cancer susceptibility of modern humans.

For the human PVs shared with other species, they were mostly present in the species distant to the humans in the phylogenetic tree, whereas few human PVs shared with non-human species in Primates. This suggests that the same human PVs could be more deleterious in primates that they could be largely eliminated by evolution selection during the long period after their separation from the humans. Currently, there are no established theories to explain why there are so many human PVs shared in distant species in Fish and Aves, although several hypotheses have been proposed in trying to explain the observation. A study compared multiple human deleterious mutations, including several mutations in BRCA, shared with mice [67]. Upon evaluating multiple hypotheses including the “Founder effect,” “Fixations of slightly deleterious mutations,” “Relaxed selection on late-onset phenotypes” and “Compensatory changes,” they were in favor of the “compensation theory,” which states that “compensatory mutations at other sites of the same or a different protein render the deleterious mutations neutral,” to explain the sharing of human deleterious mutation with distant species. The compensation theory may also be used to explain the sharing of human MMR PVs in the species distant from the humans. Regarding the MMR PVs shared between modern human and ancient humans, a possible explanation is that the short timing of 10,000 years may not be long enough to allow evolution selection to eliminate them. These PVs likely deemed to be pathogenic in modern humans and ancient humans, as supported by rich evidence from modern humans.

MMR PVs in colorectal cancer account only for around 3%. Our own MMR study in 33,998 Chinese consisting of 23,938 cancer and 10,060 non-cancer cases also observed a lower PV prevalence of 1.6% in the cohort [68]. The rarity of MMR PVs could affect the representation of our study. In order to minimize the possible effects, we applied three approaches: 1. We collected nearly all genomic data from the ancient humans currently available to maximize the representation of ancient human population (over 5000); 2. We included all MMR PVs in ClinVar to represent the MMR PVs in modern humans; 3. We included MMR founder mutations as the internal control, which are highly prevalence in certain populations. Applying these approaches should significantly increase the reliability of the data under the scope of MMR PVs currently available. While the inclusion of more MMR PVs data available in the near future can further improve the representation, we consider that the basic conclusion from our current study may not change much: MMR PVs in modern humans were not originated from non-human species but from human itself.

The pathogenic variants are deemed to be deleterious; therefore, many of them must be at rare prevalence in the population by evolution suppression. The comparison of rare variants between different species be done at the population levels. While the rare pathogenic variants in humans were identified at the population level as tens of thousands of human individuals have been sequenced, it is not the case for most of the non-human species for which only limited individuals were sequenced. Therefore, a possibility cannot be ruled out that the absence of human pathogenic variants in non-human species might be due to the lack of population-level genomic data. While the high rate of BVs and VUS sharing between the humans and non-human species and the higher rate of human pathogenic variants in non-primate species provided reasonable controls to partially address the lack of human pathogenic variants in non-human species, it is unlikely in the foreseeable near future to have extensive rare variation data from the non-human species, particularly these in the Primates, at populational levels as achieved in the humans, due to the ethic restriction and high cost. Therefore, the limitation needs to consider the lack of human pathogenic variation in non-human species as observed from our current study.

Because of the low quality of the ancient DNA, annotation procedures for the ancient variant data were less stringent than those from fresh DNA samples [34]. We used the aligned BAM files generated by the original laboratory instead of the raw sequence data to extract variant data, as it provided a matched quality control to ensure the high quality of the variant data. Only the variants identified by the reference genome sequences of hg19, GRCH37 or hs37d5 were collected, as these three reference genome sequences were considered the same in autosomal chromosomes where the MMR genes are located. The mapDamage program was used in the following steps to locate and eliminate the variants possibly generated due to the damaged ancient DNA. The process ensured the consistency of the variant data from different sources for our study.

A limitation of our study is the lack of MMR PV data from African population. This may affect the data interpretation for the arising time of MMR PVs in recent human history. Further investigation with more ancient human data should also provide more evidence to support the conclusion that human MMR PVs were mostly arisen in the past 10,000 years.

## Conclusion

Data from our study indicate that MMR PVs in modern humans were arisen within the recent human evolutionary history.

### Supplementary Information

**Additional file 1: Fig. S1. **Locations of PVs in functional domain of each MMR gene. The PVs below each protein schematic are all the PVs retrieved from ClinVar, and the PVs above are the PVs shared with other vertebrates.

**Additional file 2: Table S1.** Sources of the origin data used in the study.

**Additional file 3: Table S2.** MMR PV list. **A** Human MLH1 PVs; **B** Human MSH2 PVs; **C** Human MSH6 PVs; **D** Human PMS2 PVs.

**Additional file 4: Table S3.** Human MMR PVs shared with other species. **A** MLH1 PVs; **B** MSH2 PVs; **C** MSH6 PVs; **D** PMS2 PVs.

**Additional file 5: Table S4.** List of MMR founder PVs identified by haplotype analysis.

**Additional file 6: Table S5.** MMR PVs in ancient humans. **A** Summary of MMR PVs identified in ancient human. **B** MLH1 PVs in ancient humans; **C** MSH2 PVs in ancient humans; **D** MSH6 PVs in ancient humans; **E** PMS2 PVs in ancient humans.

## Data Availability

All data generated or analyzed during this study are included in this published article and its supplementary information files.

## Abbreviations

ATP: Adenosine triphosphate
BP: Before present
BV: Benign and likely benign variant
ENA: European Nucleotide Archive
MHC: Major histocompatibility complex
MMR: Mismatch repair
NGDC: National Genomic Data Center
PHAST: Phylogenetic analysis with space/time models
PV: Pathogenic and likely pathogenic variant
VUS: Variants of unknown significance
