# Supplementary figures and images for: Evolutionary origin of germline pathogenic variants in human DNA mismatch repair genes
Source: Hum Genomics. 2024 Jan 29;18:5. doi: 10.1186/s40246-024-00573-0 (PMC10823654; doi:10.1186/s40246-024-00573-0)

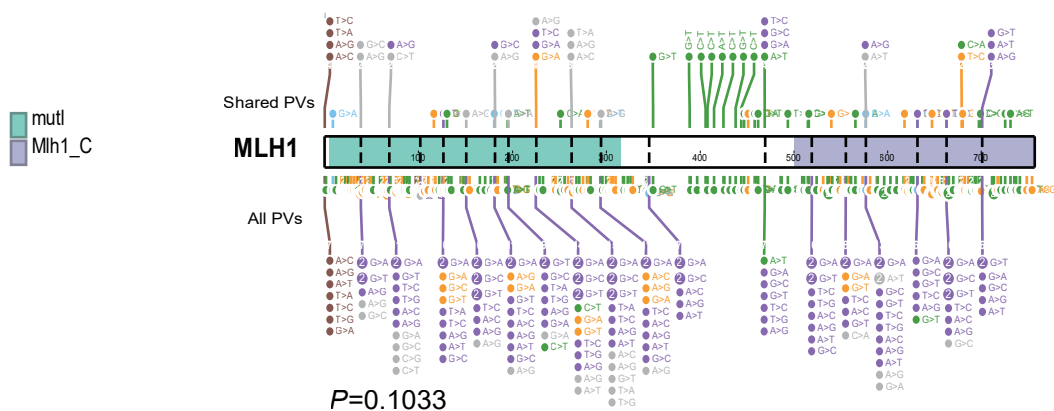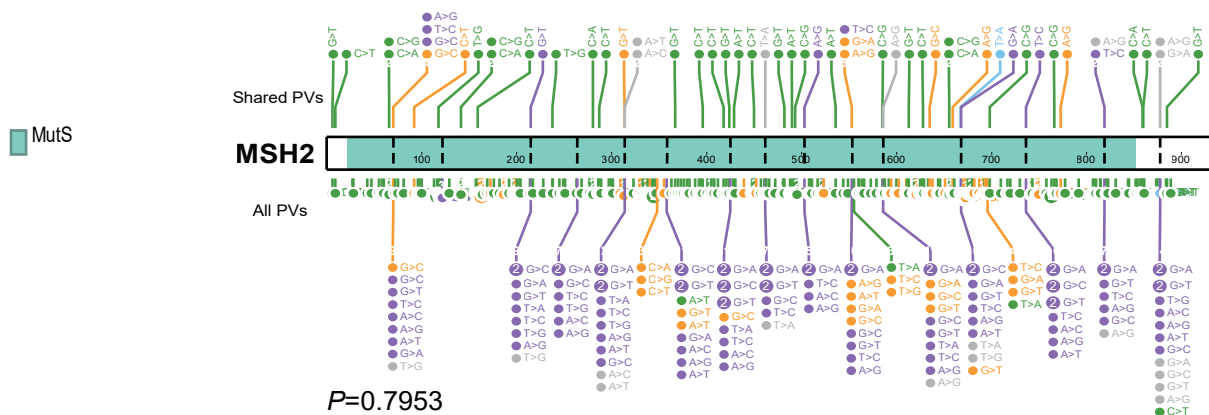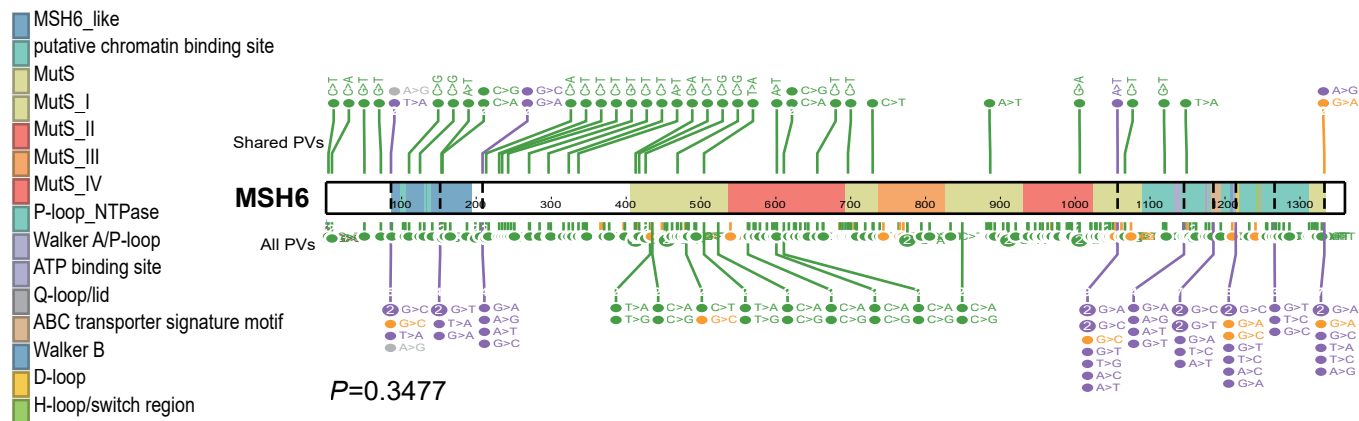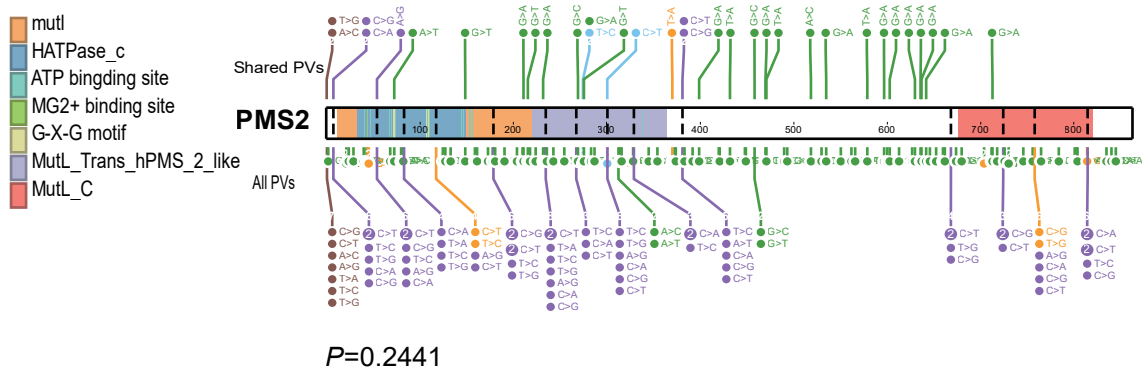

Supplement: Supplementary file 1 — Additional file 1: Fig. S1. Locations of PVs in functional domain of each MMR gene. The PVs below each protein schematic are all the PVs retrieved from ClinVar, and the PVs above are the PVs shared with other vertebrates. [file 40246_2024_573_MOESM1_ESM.pdf]
